# Multifocal White Matter Lesions Associated with the D313Y Mutation of the α-Galactosidase A Gene

**DOI:** 10.1371/journal.pone.0055565

**Published:** 2013-02-05

**Authors:** Malte Lenders, Thomas Duning, Michael Schelleckes, Boris Schmitz, Sonja Stander, Arndt Rolfs, Stefan-Martin Brand, Eva Brand

**Affiliations:** 1 Internal Medicine D, Department of Nephrology, Hypertension and Rheumatology, University Hospital Muenster, Muenster, Germany; 2 Department of Neurology, University Hospital Muenster, Muenster, Germany; 3 Institute for Sports Medicine, Molecular Genetics and Cardiovascular Disease, University Hospital Muenster, Muenster, Germany; 4 Department of Dermatology, University Hospital Muenster, Muenster, Germany; 5 Albrecht-Kossel-Institute for Neuroregeneration, University of Rostock, Rostock, Germany; Baylor Research Institute, United States of America

## Abstract

White matter lesions (WML) are clinically relevant since they are associated with strokes, cognitive decline, depression, or epilepsy, but the underlying etiology in young adults without classical risk factors still remains elusive. Our aim was to elucidate the possible clinical diagnosis and mechanisms leading to WML in patients carrying the D313Y mutation in the α-galactosidase A (GLA) gene, a mutation that was formerly described as nonpathogenic. Pathogenic GLA mutations cause Fabry disease, a vascular endothelial glycosphingolipid storage disease typically presenting with a symptom complex of renal, cardiac, and cerebrovascular manifestations. We performed in-depths clinical, biochemical and genetic examinations as well as advanced magnetic resonance imaging analyses in a pedigree with the genetically determined GLA mutation D313Y. We detected exclusive neurologic manifestations of the central nervous system of the “pseudo”-deficient D313Y mutation leading to manifest WML in 7 affected adult family members. Furthermore, two family members that do not carry the mutation showed no WML. The D313Y mutation resulted in a normal GLA enzyme activity in leukocytes and severely decreased activities in plasma. In conclusion, our results provide evidence that GLA D313Y is potentially involved in neural damage with significant WML, demonstrating the necessity of evaluating patients carrying D313Y more thoroughly. D313Y might broaden the spectrum of hereditary small artery diseases of the brain, which preferably occur in young adults without classical risk factors. In view of the existing causal therapy regime, D313Y should be more specifically taken into account in these patients.

## Introduction

White matter lesions (WML) are clinically relevant since they are associated with a variety of neurological disorders, e. g. strokes, cognitive decline, depression, or epilepsy [1]. WML are frequently documented in brain MRI in elderly subjects. The most prominent risk factors are age and essential hypertension, followed by the remaining classical cardiovascular risk factors [2]. WML may also be detected in younger adults without typical risk factors and are occasionally associated with inflammatory, and, in particular, demyelinating diseases [3]. However, despite extensive diagnostic efforts, the underlying etiology often remains elusive in these patients.

Fabry disease (FD; Online mendelian inheritance in man (OMIM) #301500) is a X-linked (Xq22.1) inborn error of glycosphingolipid catabolism resulting from deficient α-galactosidase A activity (GLA; OMIM #300644) due to mutations in the *GLA* coding region, leading to the systemic accumulation of globotriaoslyceramide (Gb3 or GL-3) in the plasma and cellular lysosomes of vessels, nerves, tissues, and organs [4]. Without enzyme replacement therapy (ERT), life span in FD patients is dramatically shortened, generally due to heart failure, renal dysfunction and cerebrovascular disease. Ischemic strokes and WML are characteristic neurological complications of FD [5]. Although mono-organic manifestations (e. g. an atypical ‘cardiac variant’) have been described [6], a disease manifestation that is limited to WML has not yet been reported.

The first identified missense mutation leading to a so-called pseudo-deficient allele was *GLA* D313Y (Exon 6; c.937G>T), which results in decreased enzyme activity in plasma, but nearly normal activity in leukocytes [7]–[9]. Although controversially discussed, the D313Y mutation is considered as non-pathogenic by most authors and, thus, D313Y carriers are not treated with ERT [10]. In the current work, we identified an index patient with significant WML carrying D313Y. After thorough exclusion of other diseases, biochemical and molecular studies, and recruitment of 7 more affected family members, we exclusively identified D313Y potentially causing manifest WML as cerebral manifestations of FD. We, consequently, evaluated the differential impact of D313Y on clinical manifestations and concluded that D313Y might broaden the spectrum of hereditary small artery diseases of the brain which preferably occur <45 years of age and should be more specifically taken into account in patients with multifocal WML in the absence of classical risk factors.

## Materials and Methods

All investigations were performed after approval of the Medical Association of Westfalian-Lippe and the Ethical Committee of the Medical Faculty of the University of Muenster (project-no.: 2011-347-f, date of report: 07.07.2011) and written informed consent of the patients for molecular analysis and publication.

### Biochemical and Molecular Studies

Genomic DNA was isolated from leukocytes with subsequent sequencing of *GLA* exons, 30–50 bp of adjacent introns and a 700 bp genomic fragment of the regulatory *GLA* 5′-sequence.

RNA extraction from leukocytes for expression analysis was done by NucleoSpin RNA Blood-Kit (Macherey-Nagel, Dueren, GER). cDNA synthesis was accomplished with SuperScript II Reverse Transcriptase Kit (Invitrogen, Darmstadt, GER). Subsequent semi-quantitative PCR was performed with oligonucleotides for *glyceraldehyde 3-phosphate dehydrogenase* (*GAPDH*) and *GLA* amplifying fragments of 93 bp and 118 bp. Western blot analysis was performed with primary anti-GLA antibody (Shire, Berlin, GER) and secondary anti-mouse HRP-coupled antibody (GE Healthcare, Little Chalfont, UK).

*GLA* sequencing, GLA activity measurements in leukocytes and determination of lyso-Gb3 contents were performed at the University Hospital of Rostock (A. Rolfs). GLA activity in plasma was determined using 4-methlyumbelliferyl-α-D-galactopyranoside (Santa Cruz Biotechnology, Santa Cruz, USA), as described previously [11]. N-acetylgalactosamine (Santa Cruz Biotechnology, Santa Cruz, USA) was used as specific inhibitor of endogenous α-Galactosidase B activity. GLA enzyme activity was measured as nanomoles (nmol) of substrate hydrolyzed per hour (h) per mg protein or ml plasma.

### MRI Data Analysis

Lesion load on axial FLAIR sequences was determined semi-automatically by outlining the peripheral borders of WML. Lesions were marked and borders were set by local thresholding using a custom-tailored software based on Analyse-software (Brain Imaging Resource, Mayo Clinic, Rochester, MI, USA). By multiplying with the interslice distance, total volume of WML is established. Intra- and interobserver reliability was high with a weighted kappa of 0.98 and 0.93, respectively.

## Results

### Patient Characteristics and Clinical Features

**Figure 1:**
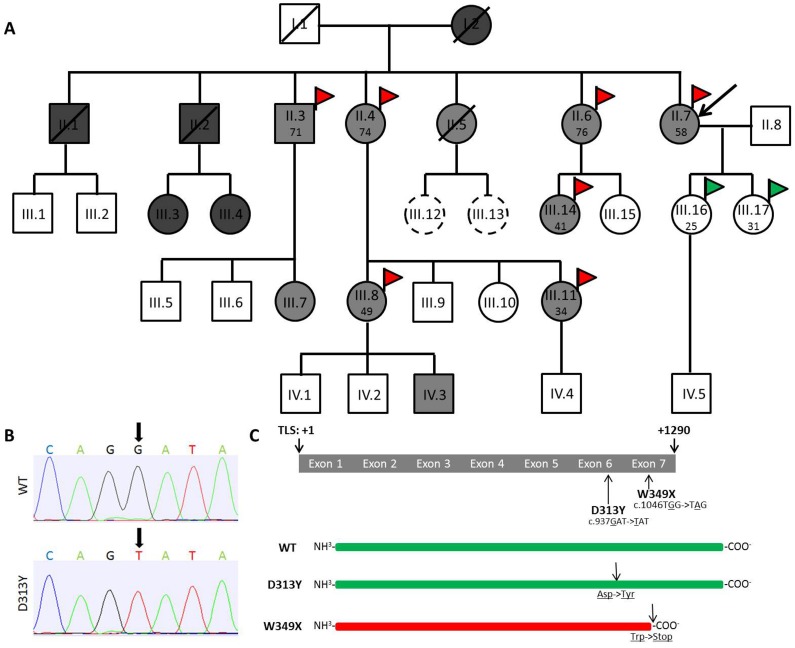
Pedigree and positions of D313Y and W349X in the *GLA* coding region. (**A**) Pedigree. (**B**) Representative chromatograms showing nucleotide substitution at position +937 (G>T) in the *GLA* coding region. (**C**) Schematic overview of the *GLA* transcript including localizations of D313Y and W349X. The pedigree shows the complete family of index patient II.7. Black arrow in A labels index patient. Squares indicate males, circles indicate females. Diagonal lines indicate deceased family members. Dark grey, light grey, and white color in squares and circles indicates W349X, D313Y, and non-carriers, respectively. Scattered circles represent not sequenced patients. Red flags indicate patients with white matter lesions (WML) seen in magnetic resonance imaging (MRI), green flags indicate control patients without WML. Patient’s age at MRI is given in years. D313Y results in single amino acid substitution at position +313, leading to a conversion of aspartate (Asp) to tyrosine (Tyr). W349X results in a c-terminal truncated GLA protein, due to a conversion of tryptophan (Trp) to a stop-codon. A: Adenine; C: Cytosine; G: Guanine; T: Thymine; TLS: translational start side; WT: wild-type GLA without any coding mutations.

Index patient II.7 specifically reported burning pain and paraesthesia in hands and feet for several years. The patient presented to our hospital for possible diagnosis of FD, as two brothers (II.1 and II.2) already developed classical FD phenotypes (Figure 1). Subject II.1 died at 38 years of age from myocardial infarction, subject II.2 at the age of 53 from renal failure. Both brothers were hemizygous carriers of the W349X mutation and showed typical FD manifestations. We screened the family and identified at least 10 members with the D313Y mutation (Figure 1). In contrast to W349X, which leads to a truncated inactive GLA protein and severe FD manifestations, D313Y only resulted in decreased plasma GLA activities (Figure 1 B+C). However, FD-typical clinical symptoms of subject II.7 prompted us to perform appropriate diagnostic procedures including biochemical and genetic analyses.

**Figure 2:**
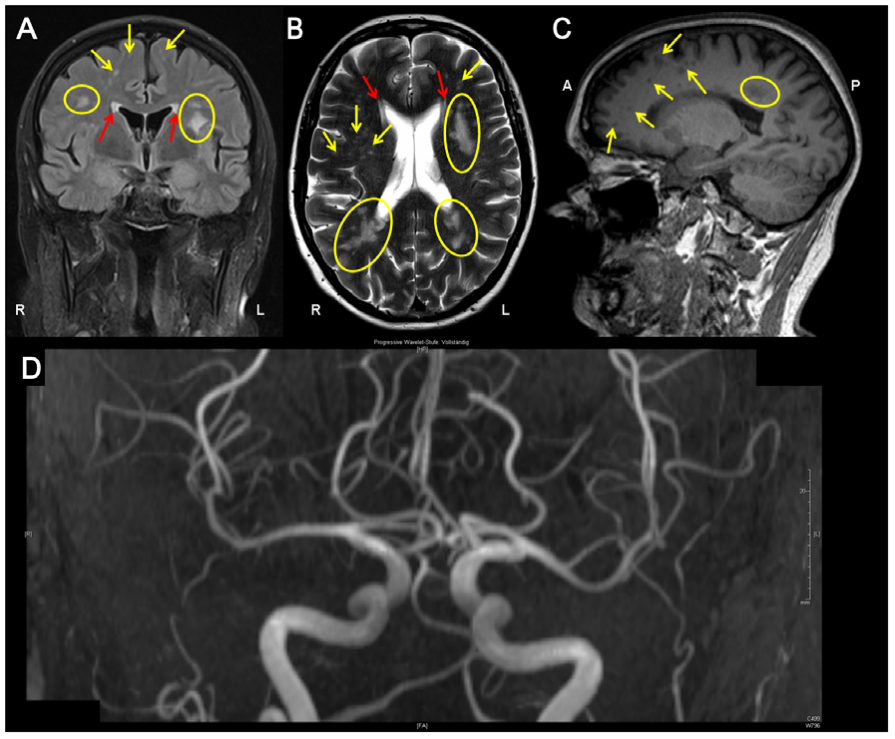
FLAIR- (A), T2- (B) and T1- (C) MR images of index patient II.7 showed widespread, punctuated (arrows) and confluent (yellow circles) WML from periventricular (yellow) to subcortical (red) without gadolinium enhancement. Lesions were associated with “black holes” in T1-weigthed images (C) as a surrogate of severe demyelination and axonal injury. MR-angiography (D) showed no signs of cerebral vasculitis or intracranial arteriosclerosis.

The neurological examination on admission was normal. Nerve conduction velocity and somatosensory, visual and motor evoked potentials showed normal latencies. MRI of the brain revealed multiple, disseminated, T2 hyperintense, partly confluent lesions from periventricular to subcortical without gadolinium enhancement (Figure 2). Spinal cord MRI was unrevealing. Two cerebrospinal fluid (CSF) analyses (4 and 7 month after first symptoms) including immunotyping by flow cytometry and biomarkers for dementia (beta-amyloid(1–42), total tau, and hyperphosphorylated tau) were normal, CSF-specific oligoclonal bands were absent (Table 1). Manual CSF cytology did not reveal any malignant cells. DNA polymerase chain reactions in CSF for herpes simplex virus type 1 and 2, varicella zoster virus, JC virus, as well as mycobacterium tuberculosis complex and *Tropheryma whipplei* were negative. Antibody titers against measles and herpesviridae were not elevated. We observed a moderate CD4 (462/µl; normal range 500–1200/µl) lymphopaenia, while the remaining blood count (including total leukocyte count) was unsuspicious. Renal and liver function tests as well as homocysteine level were normal. Serological testing for borreliosis, syphilis and HIV-1/2 infection were unrevealing. Angiotensin-converting enzyme in serum was normal (42 U/l; normal range 15–80 U/l). Serum antibodies against the aquaporin-4 water channel, thyroid peroxidase, thyreoglobulin, glutamic acid decarboxylase, GQ1b and onconeural antibodies (anti-amphiphysin, anti-Ri, anti-Yo, anti-Hu, anti-CV2/CRMP5, anti-Ma2/Ta, anti-NMDA, LGI-1, GAD) as well as anti-cardiolipin immunoglobulin were all negative. A screen for antibodies against extractable nuclear and anti-nuclear antigens was negative. Three long-term blood pressure measurements were normal (<130/80 mmHg) with nocturnal dipping. Arylsulfatase A activity and serum very long-chain free fatty acid levels were within normal ranges. Analysis of Notch3 mutations, consistent with cerebral autosomal dominant arteriopathy with subcortical infarcts and leukoencephalopathy (CADASIL), was negative. Cerebral MR- and catheter angiography was normal with no evidence for vasculitis. Although no angiokeratomas were found, a skin biopsy was done that showed a normal lipid content. However, intraepidermal nerve fiber (IENF) density was reduced (index patient: 6 IENF/mm; normal: >9 IENF/mm; Supporting Information S1), and along with the typical clinical presentation a small-fiber neuropathy was diagnosed. Transesophageal echocardiogram, renal ultrasound and urine analysis as well as ophthalmological and dermatological examination did not show other typical organ manifestations of FD. In conclusion, the diagnostic work-up provided no indication of inflammatory, neoplastic, metabolic, degenerative or congenital diseases. Of note, no classical risk factors of WML were present.

**Table 1:** Exclusion of risk factors and elicitors for white matter lesions in index patient II.7.

	sample/application	diagnostics/risk factors	results
**Neurologic**	cerebrospinal fluid	beta-amyloid(1–42), total tau, hyperphosphorylated tau, malignant cells, oligoclonal bandsantibodies against: herpes simplex virus type 1 and 2, varicella zostervirus, epstein barr virus, treponema pallidum, JC virus, borreliaburgdorferi, mycobacterium tuberculosis, tropheryma whipplei, measlesPCR: herpes viridae, JC virus, mycobacterium tuberculosis	normal
	blood	borrelia burgdorferi, treponema pallidum, HIV-1/2, aquaporin-4 waterchannel, thyroid peroxidase, thyreoglobulin, glutamatic acid decarboxylase,GQ1b, anti-cardiolipin immunoglobulin, angiotensin-converting enzymeonco-neural antibodies: anti-amphiphysin, anti-Ri, anti-Yo, anti-Hu,anti-CV2/CRMP5, anti-Ma2/Ta, anti-NMDA, LGI-1, GADCADASIL* (*Notch3* sequencing)	normal
		arylsulfatase A activity, very long chain fatty acid levels	
	MR-angiography, catheter angiography	cerebral vasculitis	normal
	skin biopsy	small-fiber neuropathy	reduced IENF+
**Cardiac**	24 h-blood pressure monitoring, PWV#,fatty acid metabolism, ECG##	arterial hypertension, arteriosclerosis, hyperlipidemia	normal
**Renal**	serum	eGFR++, proteinuria/albuminuria,	normal
	ultrasonography	renal morphology	normal

* cerebral autosomal dominant arteriopathy with subcortical infarcts and leukoencephalopathy;

+ intraepidermal nerve fiber;

# pulse wave velocity;

## electrocardiogram,

++ estimated glomerular filtration rate (Modification of Diet in Renal Disease [MDRD] formula; Chronic Kidney Disease Epidemiology Collaboration [CKD-EPI] formula).

Two cerebrospinal fluid (CSF) analyses after 4 and 7 months after first symptoms were performed.

**Figure 3:**
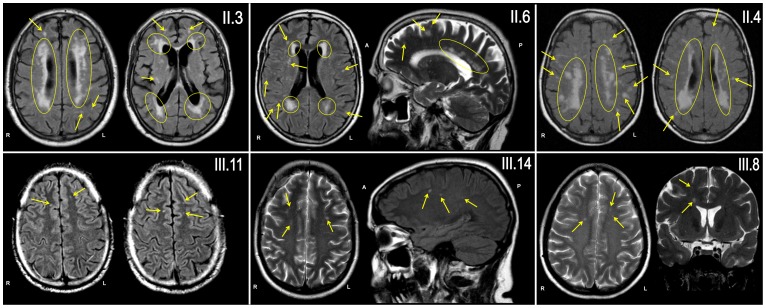
Punctuated (arrows) and confluent (yellow circles) WML were present on MR images (axial and sagittal FLAIR- and T2-sequences) of all six examined family members, all carrying D313Y. Only patient II.3 had mild cardiovascular risk factors (treated arterial hypertension). Extent and lesion load were age-related, but WML were already present in young family members without any vascular risk factor (patient III.8, III.11 and III.14; 49, 34 and 41 years of age, respectively).

**Figure 4:**
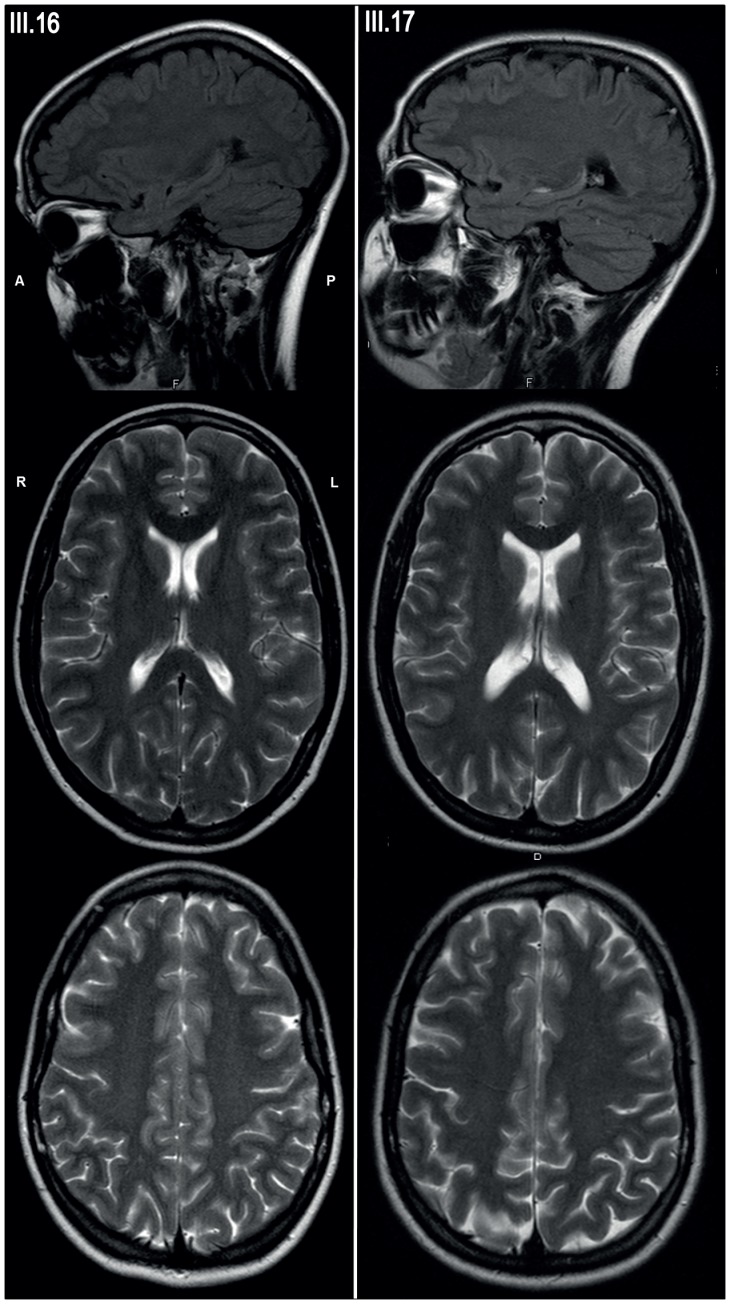
MR images of family members that were negative for D313Y did not show any white matter changes. Upper row: Sagittal FLAIR-sequence; median and lower row: Axial T2-weighted sequences of subject III.16 and III.17.

Patients II.3, II.4, II.6, III.7, III.8, III.11 and III.14, all of which carried GLA D313Y, also underwent detailed clinical examinations (Table 2). Subjects II.3 and II.7 presented with unspecific “cardiac dysfunction”. Only patient II.3 had mild cardiovascular risk factors (well-treated arterial hypertension and well-treated hyperlipidemia). To exclude cerebral manifestations, brain MRI were performed in subjects II.3, II.4, II.6, II.7, III.8, III.11 and III.14. Although no FD-specific organ manifestations were found, all patients showed different extents (lesion volumes ranged from 8.1 ml to 42.9 ml; mean 23.4 ml ±12.9 ml) of multifocal WML (Figure 3). Localisation of WML were primarily subcortical with punctuate lesions (patient II.6), but showed also confluent, periventricular involvement (patient II.3). MR images of patient II.3 showed a severe leukoencephalopathy with confluent cortical and subcortical lesions and large lesion load (42.9 ml). Overall, lesion loads were age-related, but WML were already present in young family members without any vascular risk factor (III.8 [49 y], III.11 [34 y], and III.14 [41 y]). Brain MRI was also performed in two family members that do not carry the GLA D313Y mutation. These controls (III.16 [25 y], III.17 [32 y]) showed no WMLs (Figure 4). Gadolinium enhancement was not seen in any of the cases.

**Table 2:** Summary of investigated parameters and abnormalities as typical for FD.

			Laboratory FD parameters						FD typical abnormalities		
				Enzyme activity in							
Patient	Sex	Age	CDS mutation	Plasma[nmol MU/h/ml]	Leukocytes[nmol MU/h/mg]	Lyso-Gb3[ng/ml]	GLA RNAexpression	GLA expression in plasma	Renal	Cardiac	Neurologic
II.3	m	71	D313Y	**2.39**	78	0.83	yes	yes	no	no	**WML**
II.4	f	74	D313Y	**5.67**	83	0.41	yes	yes	no	no	**WML**
II.6	f	76	D313Y	**0.60**	161	0.82	yes	yes	no	no	**WML**
II.7	f	58	D313Y	**3.21**	38	0.61	yes	yes	no	no	**WML**
III.7	f	38	D313Y	**4.89**	55	1.26	n.d.	yes	no	no	n.d.
III.8	f	49	D313Y	**5.83**	n.d	n.d.	yes	yes	no	no	**WML**
III.11	f	34	D313Y	n.d.	n.d.	1.82	n.d.	n.d.	no	no	**WML**
III.14	f	41	D313Y	**0.10**	79	0.75	yes	yes	no	no	**WML**
III.16	f	25	no	9.14	57	n.d.	yes	yes	no	no	no
III.17	f	31	no	10.1	64	n.d.	yes	yes	no	no	No

Renal parameters: ultrasonography, albumin/creatinine ratio, determination of eGFR. Cardiac parameters: echocardiography, cardiac magnetic resonance imaging (MRI) and electrocardiogram. Neural/cerebral parameters: MRI, neurography and neuropsychologic diagnostics. Reference values: plasma activity >8.0 nmol MU/h/ml; leukocyte activity >33 nmol MU/h/mg protein; lyso-Gb3<1.84 ng/ml. n.d.: not determined; WML: white matter lesions. Abnormal findings are highlighted in bold.

### Genetic and Biochemical Analysis

Re-sequencing of all seven *GLA* exons and adjacent 5′- and 3′-exon-intron-boundaries confirmed the exclusive presence of D313Y in exon 6 in all affected subjects (Figure 1, Table 2). GLA enzyme activities in leukocytes were in the normal range, or at the lower limit, but GLA activities in plasma were decreased (Table 2). Measurements of lyso-Gb3 content in blood plasma, as an additional marker for FD, were in the normal range (Table 2). To check whether GLA expression is affected by mutations within the 5′-regulatory region of *GLA*, we resequenced 700 bp of the upstream regulatory sequence, including the 5′-flanking UTR and did not detect any genetic variation. To exclude that low plasma GLA activity might result from decreased mRNA expression level or absent GLA enzyme in plasma, we performed PCR and western blot analysis (Supporting Information S1). All tested subjects showed considerable GLA mRNA expression in leukocytes and GLA protein in plasma (Table 2). Interestingly, even when CSF analyses were normal in index patient II.7, CSF-GLA activities were increased (215 pmol MU × h^−1^ × ml^−1^) compared to a healthy control group (mean: 151±22 pmol MU × h^−1^ × ml^−1^; P-value: 0.03).

## Discussion

The widespread availability of MRI has resulted in an increased recognition that WML are common incidental findings in elderly individuals >65 years of age (up to 97%), but have rarely be seen as early as in the third and fourth life decades [12]. Although the clinical relevance of such MRI findings primarily depends on the respective etiology, even incidentally discovered WML are frequently reported to be associated with various neurological symptoms, e.g. progressive cognitive and neurobehavioral deficits, gait and balance disturbances, epileptic seizures, or depression [13]–[16]. In general, WML show a strong correlation with a wide range of neurodegenerative and neuropsychiatric disorders, independent of other risk factors [17]. Specific treatment is available for some of the underlying causes, which effectively could modify symptoms and prognosis, e.g. some metabolic and inflammatory disorders. Furthermore, the cerebral lesions are often irreversible, and thus the underlying etiology is particularly important to consider. However, in clinical practice, uncovering the underlying etiology of WML in adults without cardiovascular risk factors remains dissatisfying in most of the cases. There are many different causes of WML, which can occur at all ages, be progressive or static, and be genetically determined or acquired. The diagnostic workup is complicated as many different analyses have to be performed, at high financial costs as well as emotional stress and often with disappointing results [18].

With more widespread use of neuroimaging, neurologists will increasingly be confronted with WML in younger adults. In recent years, a considerable number of new sporadic or hereditary small artery diseases of the brain have been detected which preferably occur <45 years of age [3]. FD is one of those hereditary diseases that can cause cerebral vasculopathy. Apart from macroangiopathic changes, FD is frequently associated with early microangiopathic brain alterations with progressive WML [5]. However, the typical FD symptom complex includes further manifestations such as cornea verticillata or angiokeratoma, renal or cardiac manifestations, strokes and peripheral neuropathy [4]. Of note, strokes are highly important for Fabry diagnosis, because they often occur before FD is readily diagnosed and in absence of other clinical events [19]. Although most patients present with the classical phenotype, “variant” forms with prominent cardiac or renal manifestations have been described [20]. Whether these mono- or oligo-organic phenotypes are associated with specific mutations remains unclear. In fact, efforts to associate genotype with clinical phenotype have been largely unsuccessful [4].

In the current study, we report on a family carrying the GLA D313Y mutation and being affected by a potentially exclusive neurologic manifestation of the CNS with multifocal WML, in the absence of other FD-specific symptoms. The diagnosis was further supported by the reduced intraepidermal nerve fiber density in the index patient (small-fiber neuropathy), which is also known as a frequent and early manifestation of FD. D313Y resulted in normal GLA enzyme activities in leukocytes and severely decreased activities in plasma. There is still an ongoing discussion whether D313Y is a causal GLA mutation of FD or just leading to a “pseudo-deficiency”. Recent case series or studies reported an association of D313Y with other typical FD manifestations, e.g. peripheral neuropathy, hypertrophic cardiomyopathy, renal failure, or stroke [21]–[25]. However, most authors considered the D313Y mutation as non-causal. Thus, as a consequence, to date almost all D313Y-carriers are not treated with ERT [10]. Other studies support our results and found also primarily neurological organ manifestations in patients carrying GLA D313Y. In this respect, a recent prospective study including 625 patients with cerebral ischemia aged between 18 and 55 reported that GLA D313Y was associated with cryptogenic stroke [25]. Recent reports point to an association of particular mutations with a” late-onset” or “intermediate” type of FD, e.g. N215S, A143T or F113C [26]–[28]. In this regard, the authors concluded that mutations that were formerly assumed as non-causal but nonetheless showed variable FD symptoms might be considered as “predisposing polymorphisms” (e.g. R118C or E66Q). In view of the above mentioned studies, this could particularly be the case with the D313Y mutation causing a CNS involvement. It should, however, be noted that our findings could still represent a clinical coincidence. Thus, further studies are necessary to confirm a causal relationship between the D313Y mutation and cerebral manifestations.

The significant difference of enzymatic activity in leukocytes and plasma caused by the change of aspartic acid to threonine at position 313 is most likely due to a functional intolerance to blood plasma neutral pH conditions. This obviously results in a profound decrease of enzymatic GLA activity in plasma [9]. Additionally, this effect seems to be irreversible. Once in contact with a neutral or basic pH environment D313Y remains inactive, even if transferred to optimal pH (unpublished data). The GLA substrate Gb3, also known as CD77, has been shown to act as a cell surface receptor in apoptotic signaling triggered not only by Shiga toxin and Shiga-like toxins (vero toxin), but also by Gb3/CD77 antibodies [29].

If extracellular GLA activity is involved in inactivation of CD77 or its removal from the cellular surface, then a decrease of extracellular GLA activity could lead to an increased initiation of apoptosis. If so, the occurrence of abundant WML and the mild FD symptoms in D313Y carriers points to a higher susceptibility of neural tissues to this possible pathomechanism. To strengthen the hypothesis, GLA should be further analyzed in appropriate studies as a potential extracellular regulator of CD77.

From the clinical point of view, the most appropriate time to start evaluating and follow-up the neurological manifestations of D313Y carriers, or whether and when starting treatment with ERT, remains to be investigated and should be based on more clinical data. We, therefore, decided to perform a follow-up MRI within 6 months and started a symptomatic treatment of the neuropathic pain of the index patient with pregabaline. If the neuropathic pain does not respond to appropriate medication, ERT should be suggested, in particular with regard to the excellent ERT response on neuropathic pain in appropriate studies [30]–[32]. In case of a significant increase of WML an effective anti-platelet agent such as clopidogrel should be considered as an appropriate therapeutic option.

### Conclusion

In conclusion, our results provide evidence that GLA D313Y could be involved in neural damage with consecutive WML, demonstrating the necessity of evaluating patients carrying D313Y more thoroughly. D313Y might broaden the spectrum of hereditary small artery diseases of the brain which preferably occur in young adults. In view of the existing causal therapy regime (ERT), D313Y should be more specifically taken into account in patients with multifocal WML in the absence of classical risk factors.

## Supporting Information

Supporting Information S1 The file contains supplemental methods and three supporting figures. Figure S1: Skin biopsy of index patient II.7. Lipid content was normal and intraepidermal nerve fiber density was slightly reduced. Subepidermal and intraepidermal (arrow) PGP 9.5 positive cutaneous nerve fibers. Magnification 400-fold. Figure S2: *GLA* expression in patients’ leukocytes. Semi-quantitative PCR was performed with RNA samples extracted from leukocytes. *GPDH* was used as loading control. All tested patients showed *GLA* mRNA expression in leukocytes. Figure S3: GLA protein expression in plasma. Western-blot analysis was performed with 20 µg of total plasma protein. All tested patients showed GLA protein expression in plasma. GLA protein size: 53 kDa.
(DOC)
