# Supplementary material for: Multifocal White Matter Lesions Associated with the D313Y Mutation of the α-Galactosidase A Gene
Source: PLoS One. 2013 Feb 5;8(2):e55565. doi: 10.1371/journal.pone.0055565 (PMC3564750; doi:10.1371/journal.pone.0055565)
Supplement: Supporting Information S1 — The file contains supplemental methods and three supporting figures. Figure S1: Skin biopsy of index patient II.7. Lipid content was normal and intraepidermal nerve fiber density was slightly reduced. Subepidermal and intraepidermal (arrow) PGP 9.5 positive cutaneous nerve fibers. Magnification 400-fold. Figure S2: GLA expression in patients’ leukocytes. Semi-quantitative PCR was performed with RNA samples extracted from leukocytes. GPDH was used as loading control. All tested patients showed GLA mRNA expression in leukocytes. Figure S3: GLA protein expression in plasma. Western-blot analysis was performed with 20 µg of total plasma protein. All tested patients showed GLA protein expression in plasma. GLA protein size: 53 kDa. (DOC) [file pone.0055565.s001.doc]

**Supporting Information S1**

**Multifocal white matter lesions associated with the D313Y mutation of the α-galactosidase A gene**

Malte Lenders^1^*; Thomas Duning^2^*; Michael Schelleckes^1^; Boris Schmitz^1,3^; Sonja Stander^4^; Arndt Rolfs^5^; Stefan-Martin Brand^3^; Eva Brand^1^

*These authors contributed equally to the manuscript.

1 Internal Medicine D, Department of Nephrology, Hypertension and Rheumatology, University Hospital Muenster

2 Department of Neurology, University Hospital Muenster

3 Institute for Sports Medicine, Molecular Genetics and Cardiovascular Disease, University Hospital Muenster, Muenster

4 Department of Dermatology, University Hospital Muenster

5 Albrecht-Kossel-Institute for Neuroregeneration, University of Rostock

**Supplemental methods:**

**Skin biopsy:**

Biopsy was immediately fixed in 4% paraformaldehyde for 2 h, and then kept in 5% sucrose overnight, buffered in 10% and 20% sucrose and after 6 h stored in liquid nitrogen. Three 40 µm cryosections were sectioned of each biopsy, thawed on microscope slides (DAKO Cytomania GmbH, Hamburg, Germany) and processed for immunofluorescence staining. In brief, sections were incubated overnight with the primary antibody, a polyclonal antibody against the neuron-specific hydrolase protein gene product 9.5 (PGP 9.5; rabbit 1:2000; Chemicon, Temecula, CA, USA) at room temperature. The sections were stained with the secondary antibody anti-rabbit-FITC (1:50; pork anti-rabbit immunoglobulin FITC, DAKO AIS, Denmark) for 1 h. The mounting medium was glycerin. Fluorescent samples were read using a Zeiss Axioplan fluorescence microscope and dermal and epidermal nerve fibers were manually focused in order to detect each nerve branch and its course as suggested by guidelines (Lauria et al. 2005). The length of the epidermis was measured with Olympus DP soft analySIS Image Processing (Version 3.2) software. Three specimens per biopsy were counted; mean value was determined and divided by the epidermal length of the biopsy. IENF density was calculated as mean number of nerve fibers per millimeter epidermal length (IENF/mm).

| 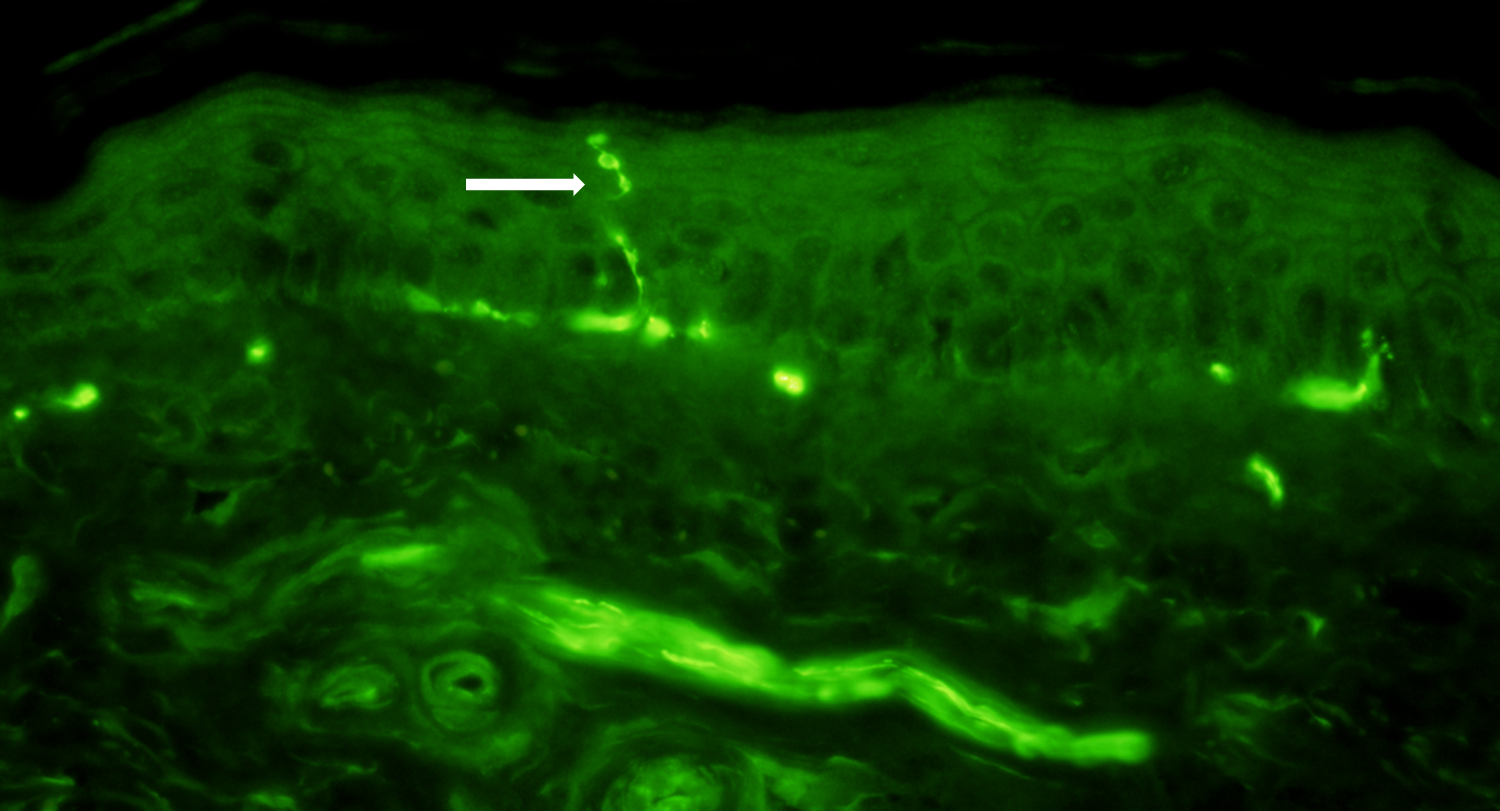 |
| --- |
| **Figure S1: Skin biopsy of index patient II.7.** Lipid content was normal and intraepidermal nerve fiber density was slightly reduced. Subepidermal and intraepidermal (arrow) PGP 9.5 positive cutaneous nerve fibers. Magnification 400-fold. |

| 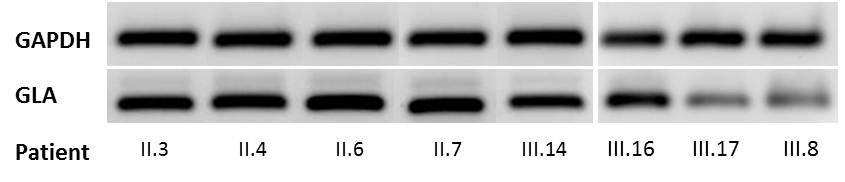 |
| --- |
| **Figure S2: *GLA* expression in patients’ leukocytes.** Semi-quantitative PCR was performed with RNA samples extracted from leukocytes. *GPDH* was used as loading control. All tested patients showed *GLA* mRNA expression in leukocytes. |

| 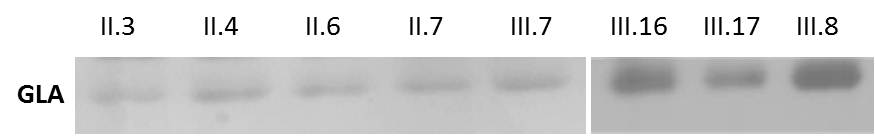 |
| --- |
| **Figure S3: GLA protein expression in plasma.** Western-blot analysis was performed with 20 µg of total plasma protein. All tested patients showed GLA protein expression in plasma. GLA protein size: 53 kDa. |

**Supplemental Reference:**

Lauria G, Cornblath DR, Johansson O, et al. (2005) EFNS guidelines on the use of skin biopsy in the diagnosis of peripheral neuropathy. *Eur J Neurol* 12: 747-758.
